# Current classification systems in slipped capital femoral epiphysis: Clinical relevance, limitations, and future directions

**DOI:** 10.1051/sicotj/2026083

**Published:** 2026-10-15

**Authors:** Clevio Desouza, Vishnu Senthil Kumar, Aravinth Thamizholi, Karthik Loganathan, Nawin Suriya Arunachalam, Ramachandran Umapathy, Pradeep Pandian Kumaramurugan

**Affiliations:** 1 Department of Orthopedics, Mahatma Gandhi Memorial Hospital, Parel Mumbai Maharashtra India; 2 Senior Assistant Professor, Department of Orthopedics, Government Royapettah Hospital, Kilpauk Medical College Chennai India; 3 Senior Assistant Professor, Department of Orthopedics, Government Stanley Medical College Chennai India; 4 Senior Assistant Professor, Department of Orthopedics, Government Stanley Medical College Chennai India; 5 Senior Resident, Department of Emergency Medicine, Government Royapettah Hospital, Kilpauk Medical College Chennai India; 6 Junior Resident, Department of Orthopedics, Government Royapettah Hospital, Kilpauk Medical College Chennai India; 7 Junior Resident, Department of Orthopedics, Government Royapettah Hospital, Kilpauk Medical College Chennai India

**Keywords:** Slipped capital femoral epiphysis, SCFE classification, Loder classification, Southwick classification, Hip preservation surgery

## Abstract

Slipped capital femoral epiphysis (SCFE) is the most common adolescent hip disorder and remains a significant cause of pain, gait disturbance, proximal femoral deformity, and early degenerative hip disease. Accurate classification of SCFE is essential for diagnosis, prognostication, treatment planning, and communication among orthopedic surgeons. Over the past several decades, multiple classification systems have been proposed based on symptom duration, physeal stability, radiographic severity, and deformity morphology. However, substantial variability exists regarding their reliability, prognostic value, and clinical applicability. This review provides a comprehensive overview of the currently available classification systems in SCFE, including temporal, Loder, Southwick, Wilson, epiphyseal tubercle (Maranho), MRI-based, and three-dimensional/statistical shape modeling classifications. Particular emphasis is placed on their historical evolution, clinical relevance, prognostic implications, limitations, and emerging role in modern hip preservation strategies. Among existing systems, the Loder classification remains the most clinically influential because of its strong association with avascular necrosis risk, while the Southwick classification continues to play a central role in quantifying deformity severity and guiding surgical decision-making. Emerging imaging-based and three-dimensional classifications have improved understanding of the rotational and multiplanar nature of SCFE and may facilitate earlier diagnosis, improved deformity characterization, and individualized treatment planning. Nevertheless, no single classification system comprehensively integrates physeal stability, deformity severity, vascular compromise, cartilage injury, and long-term functional prognosis. Current SCFE classification systems remain valuable tools in contemporary clinical practice; however, important limitations persist regarding reproducibility, prognostic accuracy, and integration with modern biomechanical concepts. Future classification frameworks may benefit from a multidomain approach integrating physeal stability, deformity morphology, vascular and biological status, cartilage involvement, and patient-specific prognostic factors, with prospective multicentre validation required before clinical adoption.

## Introduction

Slipped capital femoral epiphysis (SCFE) is the most common hip disorder affecting adolescents and represents an important cause of hip pain, gait disturbance, and long-term functional impairment in the pediatric population [1]. The condition is characterized by displacement of the proximal femoral epiphysis relative to the metaphysis through the hypertrophic zone of the physis, typically occurring during periods of rapid skeletal growth [2]. Although the exact etiology remains multifactorial, mechanical, hormonal, genetic, and metabolic factors have all been implicated in the pathogenesis of SCFE [3, 4]. Early diagnosis and timely intervention are essential, as delayed treatment may result in progressive deformity, femoroacetabular impingement, avascular necrosis, chondrolysis, and early osteoarthritis [5, 6].

Over the years, several classification systems have been developed to characterize SCFE based on symptom duration, physeal stability, radiographic severity, and deformity morphology. These classifications are intended not only to describe the disease process but also to guide treatment decisions, predict complications, and facilitate communication among clinicians and researchers [7, 8]. Among the most widely utilized systems are the temporal classification into acute, chronic, and acute-on-chronic slips [9], the Loder classification based on physeal stability [10], and the Southwick classification, which quantifies slip severity radiographically using the epiphyseal-shaft angle [9]. Additional systems incorporating radiographic morphology, three-dimensional deformity assessment, and advanced imaging modalities have also been proposed in recent years [11, 12].

Despite the widespread use of these classification systems, considerable limitations remain. Many classifications demonstrate variable interobserver reliability and limited prognostic accuracy. Certain systems focus primarily on symptom duration, whereas others emphasize mechanical deformity or risk of vascular compromise, resulting in substantial heterogeneity in clinical application. Furthermore, no universally accepted classification comprehensively integrates stability, severity, vascular risk, cartilage injury, and long-term functional prognosis. With evolving understanding of SCFE biomechanics and the increasing use of advanced imaging techniques such as magnetic resonance imaging (MRI) and three-dimensional computed tomography (3D CT), the relevance and applicability of traditional classification systems continue to be questioned.

A thorough understanding of the currently available SCFE classification systems is therefore essential for orthopedic surgeons, trainees, and researchers involved in pediatric hip preservation. Evaluating the strengths, limitations, and clinical utility of these systems may help identify gaps in current knowledge and guide future development of more comprehensive and prognostically relevant classification frameworks.

The purpose of this review is to provide a comprehensive overview of the existing classification systems in slipped capital femoral epiphysis, with emphasis on their historical evolution, clinical relevance, prognostic value, limitations, and implications for contemporary management.

## Methods

A structured literature search was performed to identify publications describing or evaluating classification systems for SCFE, as well as studies addressing the clinical relevance, reliability, prognostic value, deformity characterization, and hip preservation implications of these classifications. The databases PubMed/MEDLINE, Embase, and Scopus were searched from database inception to December 2025. The search strategy combined terms related to SCFE and classification, including “slipped capital femoral epiphysis,” “SCFE,” “slipped upper femoral epiphysis,” “classification,” “classification system,” “Loder,” “Southwick,” “Wilson,” “epiphyseal tubercle,” “Maranho,” “MRI,” “three-dimensional,” “statistical shape modeling,” “deformity,” “femoroacetabular impingement,” “hip preservation,” and “residual deformity.” Reference lists of relevant articles and review papers were also screened to identify additional publications.

Studies were considered relevant if they described an established or emerging classification system for SCFE, evaluated its reliability or clinical applicability, investigated its prognostic value, or examined the relationship between SCFE-related morphology and subsequent hip dysfunction or hip preservation treatment. Studies addressing residual proximal femoral deformity, femoroacetabular impingement (FAI), and reconstructive treatment following SCFE were also considered for the section addressing young adults after physeal closure. Publications in English involving human subjects were prioritized. Editorials, conference abstracts without sufficient methodological or clinical information, and studies unrelated to SCFE classification or its clinical implications were excluded.

Relevant studies were selected by review of titles and abstracts followed by assessment of the full text when necessary. Classification systems were included based on their historical importance, frequency of clinical use, relevance to prognosis or treatment, or contribution to contemporary imaging-based assessment of SCFE. Because this article was designed as a narrative review rather than a systematic review or meta-analysis, formal risk-of-bias assessment and quantitative synthesis were not undertaken.

### Historical evolution of classification systems in SCFE

The classification of SCFE has evolved considerably over the past several decades as understanding of the disease pathology, biomechanics, and long-term complications has improved. Early classification systems were primarily descriptive and focused on symptom duration and radiographic appearance, whereas modern systems increasingly emphasize physeal stability, deformity severity, and prognostic implications [8, 9].

One of the earliest methods of classifying SCFE was based on the temporal pattern of symptoms. Although this classification remains widely used because of its simplicity, subsequent studies demonstrated that symptom duration alone does not consistently correlate with slip severity, vascular compromise, or long-term prognosis [13, 14].

As radiographic assessment became more standardized, emphasis shifted toward quantifying the magnitude of deformity. The Southwick classification, introduced in 1967, represented a major advancement in SCFE evaluation. This system provided a more objective assessment of deformity severity and remains one of the most commonly utilized radiographic classifications today. The Southwick angle also gained importance in surgical planning, particularly in determining the need for corrective osteotomy or deformity reconstruction.

Further evolution occurred with the introduction of the Loder classification in 1993, which classified SCFE as stable or unstable based on the patient’s ability to bear weight, regardless of symptom duration [10]. This classification significantly altered clinical practice because unstable slips were shown to have a substantially higher risk of avascular necrosis (AVN) of the femoral head. The Loder system emphasized the prognostic importance of physeal stability and remains highly influential in guiding urgency of treatment and surgical strategy.

In addition to these major systems, several other radiographic and morphologic classifications have been proposed. These include assessments based on Klein’s line, posterior sloping angle, alpha angle, and three-dimensional deformity analysis. Advances in imaging modalities such as CT and MRI have further expanded understanding of SCFE morphology, cartilage injury, and early physeal changes. MRI-based classifications have shown potential in identifying pre-slip conditions and early instability before radiographic deformity becomes evident.

Despite the development of multiple classification systems, no single framework comprehensively addresses all clinically relevant aspects of SCFE, including stability, deformity severity, vascular risk, cartilage damage, and long-term functional prognosis. Consequently, current classification systems continue to evolve as clinicians seek more reliable and prognostically relevant methods for evaluating SCFE.

### Major classification systems in SCFE

Multiple classification systems have been proposed for SCFE, each attempting to characterize the disease based on symptom duration, physeal stability, radiographic severity, or deformity morphology. These systems play an important role in diagnosis, prognostication, treatment planning, and communication among orthopedic surgeons. However, each classification possesses distinct strengths and limitations, and no single system comprehensively addresses all clinically relevant aspects of SCFE. The principal existing classification systems and the clinical domains addressed by each are summarized in Figure 1.

**Figure 1:**
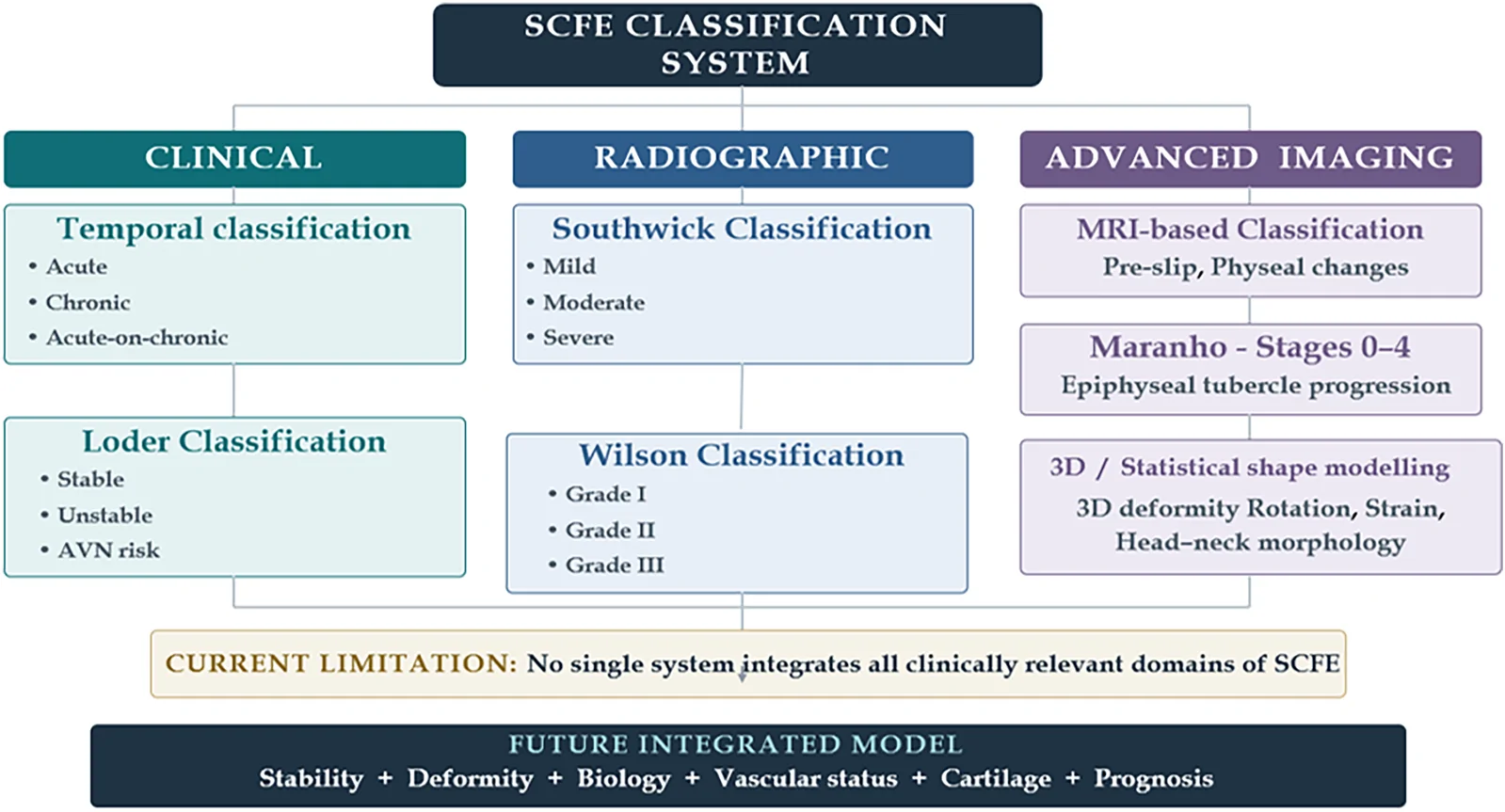
Current classification systems in SCFE and their principal clinical domains.

## Temporal classification

The temporal classification is one of the earliest and most widely recognized methods of categorizing SCFE [8]. It classifies slips into acute, chronic, and acute-on-chronic forms based on the duration of symptoms. Acute SCFE is characterized by symptom duration of less than three weeks and is often associated with sudden onset hip pain and inability to bear weight. Chronic SCFE, the most common presentation, involves symptoms persisting for more than three weeks and is typically associated with gradual onset groin, thigh, or knee pain along with progressive gait disturbance. Acute-on-chronic SCFE refers to an acute exacerbation of symptoms in a previously chronic slip.

Although this classification is simple and clinically practical, its prognostic value remains limited. Symptom duration does not always correlate with slip severity or vascular compromise, and patient-reported onset may be unreliable. Nevertheless, temporal classification continues to provide useful descriptive information during clinical assessment.

## Loder classification

The Loder classification [10], introduced in 1993, categorizes SCFE as stable or unstable based on the patient’s ability to ambulate, with or without crutches. Stable slips allow weight-bearing, whereas unstable slips prevent ambulation due to severe pain. This system has become highly influential because unstable SCFE is strongly associated with increased risk of AVN of the femoral head.

The Loder classification is clinically significant because it directly influences the urgency of treatment, surgical decision-making, and prognosis. Unstable slips often require urgent surgical stabilization and careful intraoperative management to minimize vascular compromise. Despite its widespread adoption, concerns remain regarding subjectivity in determining weight-bearing status and variability in interobserver reliability.

The Loder classification plays a key role in predicting prognosis in SCFE. In the landmark 1993 study, no cases of osteonecrosis were reported in patients with stable SCFE, while nearly half of those with unstable slips developed osteonecrosis. Subsequent studies, including systematic reviews, have consistently supported the association between instability and an increased risk of osteonecrosis [15, 16].

## Southwick Classification

The Southwick classification quantitatively assesses slip severity using the epiphyseal-shaft angle measured on frog-leg lateral radiographs [9]. Slips are categorized as mild (<30°), moderate (30°–50°), or severe (>50°). This classification provides an objective radiographic evaluation of deformity severity and is particularly useful in surgical planning.

Mild slips are commonly managed with in-situ fixation, whereas severe deformities may require corrective osteotomy or advanced hip preservation procedures. Increasing slip severity has also been associated with greater risk of femoroacetabular impingement, cartilage damage, and early degenerative changes. However, accurate measurement may be challenging in severe deformities or poorly positioned radiographs.

## Wilson classification

The Wilson classification is a radiographic grading system used to determine the severity of slipped capital femoral epiphysis based on the extent of displacement of the femoral epiphysis relative to the femoral neck, typically assessed on frog-leg lateral radiographs [17]. The degree of slippage is categorized according to the proportion of the femoral neck width involved. Mild slips (Grade I) demonstrate displacement involving less than one-third of the femoral neck width, moderate slips (Grade II) involve approximately one-third to one-half displacement, and severe slips (Grade III) are characterized by displacement exceeding one-half of the femoral neck width.

Historically, the Wilson classification played an important role in describing deformity severity and guiding clinical assessment. However, with the development of more objective angular measurements such as the Southwick slip angle, its use in contemporary clinical practice has declined. Despite this, the Wilson classification remains of historical significance and contributed to the evolution of radiographic assessment systems in SCFE.

## Other radiographic and emerging classifications

Additional classification systems have focused on radiographic morphology and mechanical factors. Parameters such as Klein’s line, posterior sloping angle, alpha angle, and three-dimensional deformity assessment have been investigated to better characterize proximal femoral morphology and predict contralateral slip risk or long-term hip dysfunction.

## Epiphyseal tubercle (Maranho) classification

More recently, Maranho et al. [11] proposed a novel radiographic staging system for SCFE based on the anatomical relationship between the epiphyseal tubercle and the metaphyseal socket, supporting the concept of a rotational mechanism of slip progression. This classification utilizes lateral hip radiographs to evaluate progressive displacement of the epiphyseal tubercle relative to the metaphysis and categorizes SCFE into five stages (Stage 0–4).

Stage 0 represents a normal relationship between the epiphyseal tubercle and metaphyseal socket without physeal widening. Stage 1 demonstrates enlargement of the metaphyseal socket with surrounding peritubercle lucency while the tubercle remains concentrically positioned. In Stage 2, eccentric displacement of the tubercle occurs while maintaining contact with the posterior metaphyseal wall. Stage 3 is characterized by posterior migration of the tubercle and metaphyseal lucency reaching the posterior cortex of the femoral neck. Stage 4 represents complete dislodgement of the epiphyseal tubercle from the metaphyseal socket, often corresponding to severe or unstable slips.

The proposed staging system demonstrated excellent intraobserver and interobserver reliability and showed strong correlation with Southwick severity grading [10] as well as moderate correlation with Loder stability classification [12]. An important advantage of this system is its potential ability to identify subtle or pre-slip stages of SCFE before significant displacement becomes radiographically apparent. Furthermore, the classification provides additional insight into the biomechanical and rotational pathogenesis of SCFE and may help identify hips at risk for progression, fixation failure, or osteonecrosis. However, despite its promising clinical implications, further validation and prospective studies are required before widespread adoption in routine clinical practice.

## 3D and statistical shape modeling classifications

Recent advances in imaging and computational modeling have introduced 3D assessment systems for evaluating deformity in SCFE [12]. Traditional two-dimensional radiographic classifications may underestimate the complex multiplanar deformities associated with SCFE, prompting increasing interest in 3D morphologic analysis using CT, MRI, and statistical shape modeling (SSM). These techniques allow detailed assessment of femoral head displacement, physeal orientation, femoral neck deformation, and regional strain patterns.

Statistical shape modeling has emerged as a promising tool for characterizing disease-specific proximal femoral morphology and quantifying deformity progression in SCFE. Chan et al. [12] demonstrated that SCFE severity is associated with progressive posterior and inferior displacement of the femoral head growth plate, increasing femoral neck deformation, and altered regional strain distribution within the proximal femur. Severe slips demonstrated substantial femoral head displacement with minimal intrinsic strain, whereas increasing strain patterns were observed at the femoral neck and greater trochanter, suggesting important biomechanical adaptations during disease progression.

3D modeling has also provided insight into the rotational and biomechanical pathogenesis of SCFE. Progressive posterior and inferior orientation of the growth plate with increasing slip severity has been consistently observed, supporting the concept that SCFE represents a multiplanar deformity rather than a simple posterior slip. These advanced imaging techniques may improve understanding of femoroacetabular impingement, cartilage injury, and residual deformity following treatment.

An important advantage of 3D classifications is their ability to provide more precise deformity characterization and assist with surgical planning, particularly in severe slips requiring corrective osteotomy or hip preservation procedures. Furthermore, early morphologic changes and abnormal strain patterns identified through computational modeling may potentially facilitate earlier diagnosis before significant radiographic displacement becomes apparent. However, despite their promising clinical applications, these techniques remain largely research-based, require specialized imaging and software, and currently lack standardized classification criteria for widespread clinical use.

Collectively, current SCFE classification systems provide valuable clinical information; however, each evaluates only specific aspects of the disease (Table 1). Consequently, integration of clinical, radiographic, and prognostic factors remains an ongoing challenge in the development of an ideal classification framework for SCFE.

**Table 1:** Major classification systems in SCFE.

Classification system	Basis of classification	Categories	Clinical utility	Limitations
Temporal classification [8]	Duration of symptoms	Acute, Chronic, Acute-on-chronic	Describes disease presentation	Poor prognostic correlation
Loder classification [10]	Weight-bearing ability	Stable, Unstable	Predicts AVN risk and urgency	Subjective stability assessment
Southwick classification [8]	Epiphyseal-shaft angle	Mild, Moderate, Severe	Quantifies deformity severity	Dependent on radiographic positioning
Wilson classification [17]	Percentage of epiphyseal displacement	Grade I–III	Historical deformity grading	Less precise than angular systems
Maranho classification [11]	Epiphyseal tubercle-metaphyseal relationship	Stage 0–4	Early instability assessment	Limited validation
MRI-based classification	Physeal edema/instability	Variable	Detects pre-slip changes	Limited availability
3D/SSM classification [12]	Three-dimensional morphology and strain analysis	Variable	Advanced deformity analysis	Primarily research-based

AVN: Avascular necrosis, MRI: Magnetic resonance imaging, SSM: Statistical shape modeling.

## Results

### Clinical relevance of classification systems

Classification systems in SCFE are not merely descriptive tools but play a critical role in guiding diagnosis, treatment strategies, prognostication, and long-term follow-up. Appropriate classification of SCFE assists clinicians in determining disease severity, assessing the risk of complications, and selecting the most suitable surgical intervention.

Among currently available systems, the Loder classification remains one of the most clinically influential because of its strong association with AVN. Stable slips generally demonstrate lower rates of osteonecrosis and favorable outcomes following in-situ fixation, whereas unstable slips are associated with significantly increased vascular compromise and poorer prognosis. Consequently, the Loder classification directly influences surgical urgency and perioperative management. Patients with unstable SCFE often require urgent stabilization and careful reduction techniques to minimize additional vascular injury.

The Southwick classification is particularly valuable in evaluating deformity severity and planning reconstructive procedures. Mild slips are frequently managed successfully with in-situ fixation alone, while moderate and severe deformities may require osteochondroplasty, corrective osteotomy, or surgical hip dislocation procedures to restore proximal femoral anatomy and reduce the risk of FAI. Increasing slip severity has also been associated with greater cartilage damage, reduced range of motion, and early degenerative changes.

Temporal classification, although less predictive prognostically, remains clinically useful in understanding disease presentation and symptom progression. Acute slips often present with severe pain and inability to ambulate, whereas chronic slips may demonstrate adaptive remodeling despite progressive deformity. Acute-on-chronic slips may indicate sudden physeal instability superimposed on longstanding deformity and frequently warrant urgent evaluation.

Emerging imaging-based and morphologic classifications have further expanded the understanding of SCFE biomechanics and residual deformity. MRI-based systems may help identify early physeal instability and pre-slip conditions before significant displacement becomes radiographically apparent. Similarly, three-dimensional imaging and statistical shape modeling provide improved assessment of multiplanar deformity, rotational abnormalities, and femoral head-neck offset changes that may contribute to postoperative impingement and osteoarthritis.

Despite their clinical utility, current classification systems remain limited by the absence of a comprehensive framework integrating stability, deformity severity, cartilage injury, vascular compromise, and long-term functional prognosis (Table 2). As a result, surgeons often rely on multiple complementary classification systems when evaluating SCFE patients. Future classification models may benefit from combining clinical, radiographic, and advanced imaging parameters to improve prognostic accuracy and optimize individualized treatment strategies.

**Table 2:** Clinical relevance of classification systems.

Classification	Primary clinical role	Prognostic evidence	Treatment relevance	Current clinical role
Temporal [8]	Describes symptom duration/presentation	Limited	Limited	Descriptive
Loder [10]	Defines physeal stability	Strong association with AVN risk	High	Widely used
Southwick [9]	Quantifies slip/deformity severity	Moderate	Important for deformity assessment	Widely used
Wilson [17]	Grades radiographic displacement	Limited contemporary prognostic evidence	Limited	Historical
Maranho [11]	Assesses epiphyseal tubercle–metaphyseal relationship	Emerging	Potential role in early disease assessment	Investigational
MRI-based	Detects physeal/cartilage abnormalities and early disease	Emerging	Potential role in early diagnosis	Emerging
3D/SSM [12]	Characterizes multiplanar proximal femoral morphology	Emerging	Potential role in deformity analysis/planning	Primarily research

AVN: Avascular necrosis, SSM: Statistical shape modeling.

## Discussion

### Reliability and limitations of current classification systems

Although numerous classification systems for SCFE have been developed, several limitations continue to affect their reproducibility, prognostic accuracy, and universal applicability. Most existing systems evaluate only isolated aspects of the disease, such as symptom duration, radiographic severity, or physeal stability, without comprehensively incorporating the complex biomechanical and biological features of SCFE.

One of the major concerns involves interobserver and intraobserver reliability. The Loder classification [10], while clinically valuable for predicting AVN risk, depends on the patient’s ability to bear weight, which may be influenced by pain tolerance, associated injuries, or physician interpretation. Consequently, the determination of stability may vary among clinicians. Similarly, radiographic classifications such as the Southwick [8] and Wilson systems [17] are influenced by patient positioning, radiographic quality, and measurement variability, particularly in severe deformities.

Another important limitation is the inconsistent correlation between classification grade and long-term clinical outcomes. Temporal classification systems [8] based on symptom duration may not accurately reflect slip severity or vascular compromise. Likewise, radiographic severity alone may not fully predict cartilage injury, femoroacetabular impingement, or future osteoarthritis. Some patients with apparently mild slips may still develop substantial functional impairment and degenerative changes.

Traditional two-dimensional radiographic classifications may also underestimate the multiplanar deformity present in SCFE. Increasing evidence suggests that SCFE involves complex rotational and three-dimensional alterations of the proximal femur, which cannot be fully appreciated on standard radiographs. Advanced imaging techniques such as MRI and CT-based statistical shape modeling have demonstrated additional morphologic abnormalities involving the femoral neck, greater trochanter, and physeal orientation.

Furthermore, many classification systems were developed before the widespread recognition of femoroacetabular impingement and hip preservation principles. Consequently, several older systems inadequately address residual deformity, impingement risk, or cartilage damage, all of which are increasingly recognized as major determinants of long-term outcomes.

Another limitation is the lack of standardized integration between classification systems and treatment algorithms. While certain systems guide general management principles, no universally accepted framework currently combines stability, deformity severity, vascular risk, and functional prognosis into a single clinically applicable model.

Despite these shortcomings, current classification systems remain valuable tools in clinical practice. However, continued refinement and validation are required to improve reproducibility, prognostic accuracy, and incorporation of modern imaging and biomechanical understanding into future SCFE classification frameworks (Table 3).

**Table 3:** Reliability and limitations.

Classification	Advantages	Major limitations
Temporal [8]	Simple and easy to apply	Poor correlation with prognosis
Loder [10]	Strong AVN association	Subjective assessment of stability
Southwick [8]	Objective angular measurement	Radiographic variability
Wilson [17]	Easy radiographic grading	Less accurate in complex deformities
Maranho [11]	Detects early instability	Limited clinical validation
MRI-based	Early disease identification	Cost and accessibility
3D/SSM systems [12]	Precise deformity analysis	Specialized software and expertise required

SSM: Statistical shape modeling.

### Applicability of SCFE classifications to residual deformity in young adults

An important limitation of existing SCFE classification systems is that most were developed to characterize the initial adolescent presentation rather than the residual deformity encountered after physeal closure. This distinction becomes particularly relevant in adolescents and young adults who present years after the initial slip with hip pain, restricted range of motion, FAI, or early degenerative changes. In this setting, historical classification according to symptom duration or physeal stability may have limited clinical relevance, while the Southwick slip angle provides only a partial description of the residual proximal femoral morphology.

The Southwick classification [9] remains useful for quantifying the magnitude of the original deformity and may provide an initial framework for assessing residual SCFE morphology. However, evidence suggests that the slip angle alone does not fully explain the mechanical consequences of the deformity. Mamisch et al. [18] demonstrated that femoral metaphyseal morphology, head-neck offset, and acetabular version influenced hip range of motion and the mechanism of impingement in patients with SCFE. In particular, reduced head-neck offset in patients with moderate slip angles could produce substantial restriction of motion, emphasizing that residual morphology may be clinically important beyond the Southwick angle alone.

This has important implications for the assessment of young adults with post-SCFE deformity. The residual deformity should be evaluated in terms of the three-dimensional relationship between the femoral head, metaphysis, femoral neck, and acetabulum, rather than being defined solely by the historical slip grade. Assessment of femoral head-neck offset, alpha angle, femoral version, acetabular morphology, and the location and extent of the metaphyseal prominence may provide additional information regarding the mechanism of impingement and the suitability of hip-preserving treatment.

The relationship between residual SCFE morphology and FAI has also influenced contemporary reconstructive strategies. A systematic review by Oduwole et al. [19] evaluating surgical treatment of FAI secondary to SCFE reported satisfactory clinical outcomes following arthroscopic osteochondroplasty, surgical hip dislocation, and open osteotomy, although the available evidence was predominantly composed of retrospective studies. The authors also highlighted that treatment decisions based solely on the Southwick slip angle may be insufficient and that correction of the cam-type metaphyseal prominence and restoration of femoral head-neck clearance are important considerations. More recent systematic review evidence similarly supports arthroscopic osteochondroplasty as a treatment option for selected patients with symptomatic FAI secondary to SCFE, with improvements in internal rotation, alpha angle, and patient-reported outcomes reported in short- to medium-term follow-up [20].

Contemporary hip-preservation assessment of cam-type morphology further supports the need to move beyond a single slip-angle measurement. Cam morphology is commonly characterized using parameters such as the alpha angle and femoral head-neck offset, and three-dimensional imaging can provide additional information regarding the location and extent of the deformity. Beaulé P et al. [21] have emphasized the importance of three-dimensional assessment of proximal femoral morphology in understanding FAI and in planning corrective procedures. In patients with residual post-SCFE deformity, these parameters may therefore complement the Southwick classification when determining whether symptoms are related to focal metaphyseal prominence, altered head-neck offset, abnormal femoral version, or a more complex multiplanar deformity.

Accordingly, the Southwick classification [9] should be regarded as a measure of slip severity rather than a complete classification of residual post-SCFE morphology. For young adults presenting after physeal closure, a clinically useful framework may require integration of historical slip characteristics with current three-dimensional femoral morphology, evidence of FAI, cartilage and labral status, range-of-motion limitation, and patient-reported symptoms. Such an approach would better align SCFE classification with contemporary hip-preservation practice and may provide more clinically meaningful guidance for reconstructive treatment.

### Future directions in SCFE classification

With advances in imaging technology, biomechanics, and hip preservation surgery, future classification systems for SCFE are likely to evolve beyond traditional symptom-based and radiographic grading methods. Increasing recognition of the complex three-dimensional nature of SCFE has highlighted the limitations of existing classifications and the need for more comprehensive, prognostically relevant models.

One of the most promising areas of development involves advanced imaging techniques such as MRI and 3D CT. MRI-based assessments may facilitate earlier diagnosis by identifying physeal edema, cartilage injury, and subtle instability before significant radiographic displacement occurs. Early detection of “pre-slip” conditions may allow timely intervention and potentially reduce progression to severe deformity. Similarly, 3D CT reconstruction and statistical shape modeling provide more accurate characterization of multiplanar deformity, femoral version abnormalities, and head-neck offset changes associated with femoroacetabular impingement.

Emerging biomechanical and computational models have further improved the understanding of SCFE pathogenesis. Statistical shape modeling and strain analysis techniques may help identify abnormal growth patterns and regions of mechanical stress that predispose to slip progression. These technologies may also contribute to individualized surgical planning and the prediction of postoperative remodeling.

Artificial intelligence (AI) and machine learning algorithms represent another potential advancement in SCFE classification. Automated image analysis may improve measurement reproducibility, reduce observer variability, and facilitate large-scale prognostic modeling using combined clinical and radiographic data. Such approaches could enable the development of predictive tools capable of estimating complication risk, slip progression, and long-term functional outcomes.

Future classification systems may also incorporate additional patient-specific variables such as skeletal maturity, body mass index, endocrinologic abnormalities, cartilage status, and vascular perfusion. Integration of these factors into a unified prognostic framework may provide more personalized treatment recommendations and improve risk stratification.

### A proposed multidomain classification framework for SCFE

Given the limitations of existing classification systems, a future SCFE classification could be developed as a multidomain framework rather than a single-dimensional grading system. Such a framework would retain the clinically useful information provided by established classifications while incorporating additional biological and morphological parameters. We propose that five complementary domains should be considered: (1) physeal stability, (2) deformity severity and morphology, (3) vascular and biological status, (4) cartilage and joint involvement, and (5) patient-specific prognostic factors (Figure 2).

**Figure 2:**
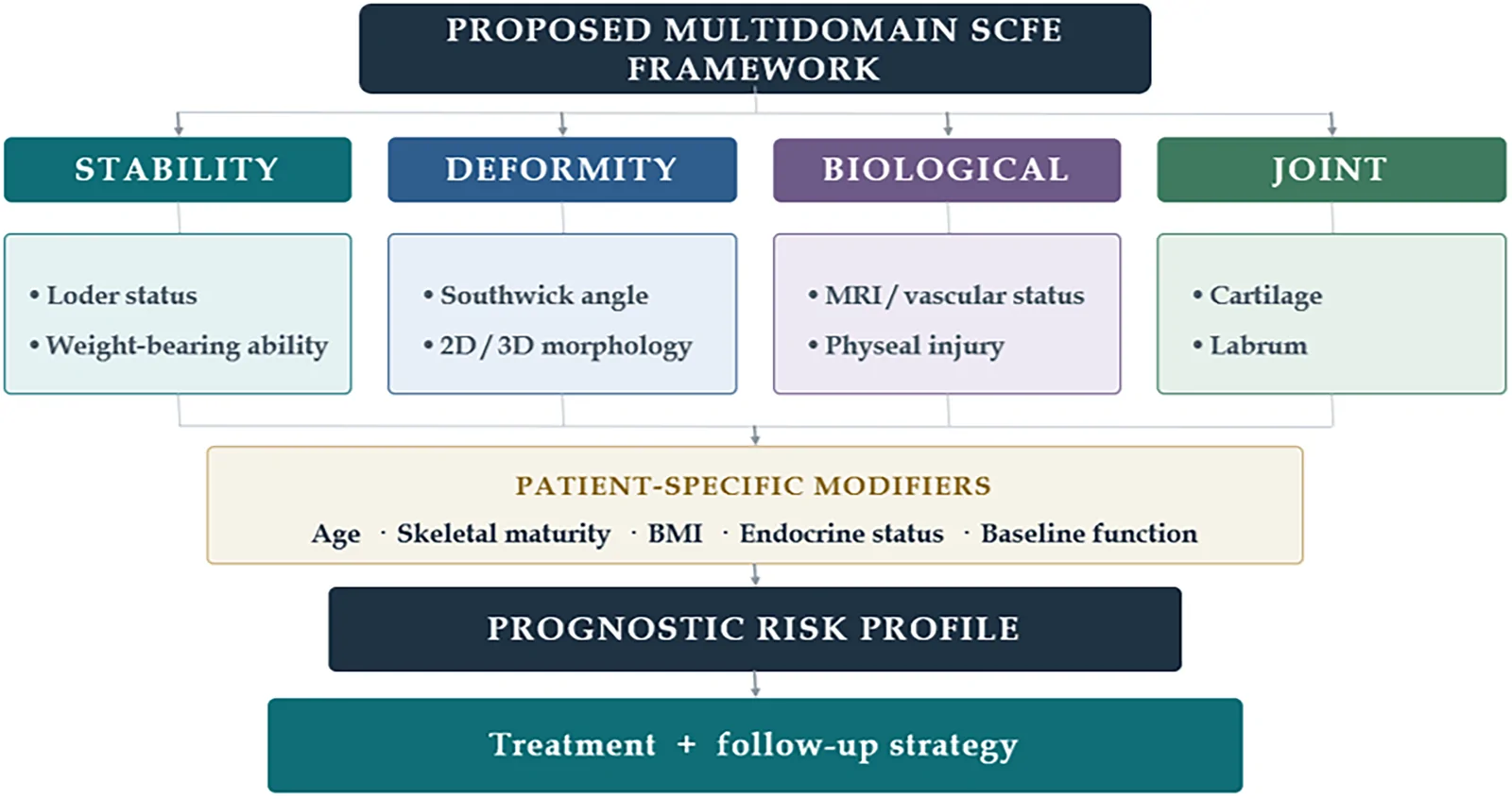
Proposed multidomain SCFE framework.

The first domain would retain the clinically important distinction between stable and unstable SCFE, reflecting the established association between instability and osteonecrosis risk. The second domain would quantify deformity using standardized radiographic or three-dimensional measurements, including Southwick slip angle, rotational deformity, femoral version, and head-neck morphology. The third domain could incorporate markers of vascular compromise and physeal or epiphyseal biological injury, potentially assessed using MRI or other validated imaging techniques. The fourth domain would characterize acetabular cartilage injury, labral pathology, and early FAI-related changes. Finally, patient-specific modifiers such as skeletal maturity, age, body mass index, endocrine or metabolic abnormalities, and baseline functional status could be incorporated to improve individualized prognostic assessment.

Rather than replacing the existing Loder or Southwick classifications, this approach could function as an integrated multidomain descriptor. For example, a patient could be described according to stability, deformity severity, biological/vascular status, joint involvement, and prognostic modifiers simultaneously. Such a system may provide greater clinical information than assigning a patient to a single categorical grade and could potentially facilitate individualized treatment selection and risk stratification.

Importantly, this proposed framework should be regarded as a conceptual model rather than a validated classification system. Future research should determine which variables demonstrate the strongest independent associations with clinically relevant outcomes, establish standardized measurement thresholds, and evaluate interobserver and intraobserver reliability. Prospective, multicentre studies could subsequently assess its ability to predict osteonecrosis, slip progression, FAI, revision surgery, patient-reported outcomes, and long-term degenerative changes. External validation in independent cohorts would be essential before such a multidomain classification could be recommended for routine clinical use.

Another important direction involves the development of classifications specifically addressing residual deformity and femoroacetabular impingement following SCFE treatment. As hip preservation surgery becomes increasingly important, greater emphasis is being placed on restoration of proximal femoral anatomy and prevention of early osteoarthritis.

Ultimately, the ideal SCFE classification system should be reproducible, clinically practical, prognostically meaningful, and capable of guiding treatment decisions while incorporating modern understanding of hip biomechanics and deformity progression. A multidomain framework integrating physeal stability, deformity morphology, vascular and biological status, cartilage involvement, and patient-specific prognostic factors may represent a more comprehensive approach than currently available single-domain classifications (Table 4). However, such a framework should remain hypothesis-generating until its individual components, thresholds, reliability, and prognostic performance have been established. Prospective multicentre studies with external validation and clinically relevant longitudinal outcomes will be necessary to determine whether an integrated classification can improve risk stratification, treatment selection, and long-term outcomes in patients with SCFE.

**Table 4:** Future directions.

Emerging concept	Potential clinical benefit
MRI-based instability markers	Earlier diagnosis and prevention
AI-assisted image analysis	Improved reproducibility
Statistical shape modeling	Personalized deformity assessment
3D CT reconstruction	Better surgical planning
Combined prognostic scoring systems	Improved treatment stratification
Cartilage and vascular assessment integration	Enhanced long-term outcome prediction

## Data Availability

No data were generated in the study.
